# Sex-Biased Networks and Nodes of Sexually Antagonistic Conflict in *Drosophila*

**DOI:** 10.1155/2013/545392

**Published:** 2013-01-22

**Authors:** Matthew E. B. Hansen, Rob J. Kulathinal

**Affiliations:** Department of Biology, Temple University, 1900 N 12th Street, Philadelphia, PA 19122, USA

## Abstract

Sexual antagonism, or conflict, can occur when males and females harbor opposing reproductive strategies. The large fraction of sex-biased genes in genomes present considerable opportunities for conflict to occur, suggesting that sexual antagonism may potentially be a general phenomenon at the molecular level. Here, we employ a novel strategy to identify potential nodes of sexual conflict in *Drosophila melanogaster* by coupling male, female, and sex-unbiased networks derived from genome-wide expression data with available genetic and protein interaction data. We find that sex-biased networks comprise a large fraction (*~*1/3) of the total interaction network with the male network possessing nearly twice the number of nodes (genes) relative to the female network. However, there are far less edges or interaction partners among male relative to female subnetworks as seen in their power law distributions. We further identified 598 sex-unbiased genes that can act as indirect nodes of interlocus sexual conflict as well as 271 direct nodal pairs of potential conflict between male- and female-biased genes. The pervasiveness of such potentially conflicting nodes may explain the rapid evolution of sex-biased as well as non-sex-biased genes via this molecular mechanism of sexual selection even among taxa such as *Drosophila* that are nominally sexually dimorphic.

## 1. Introduction

 The cooccurrence of distinct morphs—male and female—in sexually reproducing taxa continues to fascinate and perplex developmental and evolutionary biologists alike. Ranging from the subtle to the dramatic, sexually dimorphic traits are presumed to be the product of dynamically evolving genetic architectures that rapidly respond to evolutionary pressures such as sexual selection [1] (for more recent overviews, see [2]). Recent genome-wide analyses have demonstrated that sexual dimorphism is also prevalent at the level of the genome with the majority of genes expressing a male- or female-bias across a range of developmental stages [3–7]. This emerging molecular view reveals that a large fraction of the genome can be expressed in either male or female states.

Like traits, genes can possess alternative strategies depending on the sex they are expressed in. A gene that is expressed in males may provide an important and critical role in his reproductive success while the same gene, when expressed in females, may impart a similarly important but different role in her survival. Thus, fitness effects from the same locus, under different context-dependent states, may be in conflict. This particular type of antagonism, in which a single gene is expressed differently depending on the sex, has been termed, intralocus sexual conflict. Over the last two decades, intralocus sexual conflict has become an integral component of sexual selection theory providing an alternative explanation to such phenomena as the rapid evolution of reproductive traits possessing divergent functions in both males and females [8] and speciation [9–11].

Genes, however, do not work in isolation. Genetic pathways and networks demonstrate a substantive interconnectability of genes to each other and offer a direct link to competing interests. The extent of *interlocus* sexual conflict across a genome largely depends on how extensive is the linkage between male- and female-specific gene networks. In a genetic network, genes refer to epistatic interactions between different loci or nodes via edges at either a domain level (e.g., protein-protein interaction) or genetic level (e.g., transcription factor binding to regulatory domains). The emergence of genome-wide tools and resources to elucidate molecular interactions at both the genetic and protein levels has greatly increased our catalog of genome-wide interactions, making it possible to finally address interlocus (or intermolecular) conflict systematically across the entire genome [12–15].

In this paper, we generate male and female networks using transcriptome and interactome data from *D. melanogaster* to characterize network differences among male-, female-biased, and sex-unbiased genes so that we can identify potential nodes of interlocus sexual conflict, across the genome. Specifically, we describe and characterize two types of sexual conflict at the molecular level: (1) indirect conflict, which refers to sex-unbiased genes that interact with both male and female genes, and (2) direct conflict, which refers to male and female nodes that directly interact with each other. Our findings provide a first step in understanding antagonistic conflict at the molecular level, genome-wide.

## 2. Methods

### 2.1. Sex-Specific Expression in *D. melanogaster*

All expression data were obtained from the modENCODE project [16]. Specifically, separate genome-wide RNA-seq data from whole body tissue samples of two males and two females were downloaded from the Gene Expression Omnibus (http://www.ncbi.nlm.nih.gov/geo/), dataset identification GSE28078, which provided BAM files using *D. melanogaster* Genomic Assembly Release 5 as the reference genome. The *D. melanogaster* Annotation Release 5.39 gene models used were obtained from FlyBase (http://www.flybase.org/), provided as GFF files.

### 2.2. Estimating Sex Bias

The sex-bias per gene is calculated as follows. Consider a set of *k* samples of coverage data, indexed by *i* = 1,…, *k*. Let *N*_*ig*_ be the total read depth of in sample *i* over some region *g*. Likewise, define *N*_*i*_ = ∑_*g*_ *N*_*ig*_ to be the total read depth over all regions (the entire genome), and *N* = ∑_*i*_ *N*_*i*_ to be the total reads across all samples and all regions. The sample weight of sample *i* is *w*_*i*_ = *N*_*i*_/*N*. The sample average expression of a region *g* is defined as ${\overset{-}{N}}_{g}=\sum_{i}w_{i}N_{ig}$. The average total expression per sample is $\hat{N}=N/k$. The sex-bias of region *g* is defined on a log base 2 scale,

(1) bg≡log⁡2[N−gmN−gf]−B,

where the superscripts refer to male and female, and $B=\log_{2}\left[\;{\hat{N}}^{\text{m}}\;/\;{\hat{N}}^{\text{f}}\right]$ is the global average read bias between the male and female samples. *g* derives from the total CDS (one CDS isoform was randomly chosen per gene).

For each gene, we estimated sex-bias from the annotated coding sequence (CDS). Genes with zero expression across all samples were discarded, yielding 13,643 genes with a sex-bias value; 12,453 of those genes with a sex-bias value were contained in the DroID network (see below). The bias thresholds are *b* > 2 for male-biased (four-fold higher in males than females), *b* < −2 for female-biased (four-fold higher in females than males), and −2 ≤ *b* ≤ 2 for sex-unbiased nodes. Sex-bias values were capped at *b*_*g*_ = −10 and *b*_*g*_ = 10 (i.e., representing over a 1000-fold difference in expression), in order to retain non-infinite sex-bias ratios. The Kendall tau correlation coefficient between the sex-bias defined here and the sex-bias defined from a meta-analysis from the Sebida sex-bias database [17] is *τ* = 0.711.

### 2.3. Genetic Interactions in *D. melanogaster*

The gene-gene interaction network (GGIN) was downloaded from the DroID metadataset version v2012_04 [12], comprising a total of 15,254 genes and 514,325 gene-gene interactions. When assigning interactions between genes, we used two approaches based on permissive and a more conserved, or strict, criteria. The permissive approach used all 13 of the available interaction datasets from DroID—from protein-protein interactions to genetic interactions—allowing for a larger sample of genes. These included the files shown in Table 4.

A more strict approach, which formed the basis of our results, identified interaction pairs solely from six empirically-driven physical interactions (e.g., protein-interaction data derived from six experiments including yeast-two-hybrid and transcription-factor CHIP-seq analyses from the modENCODE project). These files are listed in the left column of Table 4.

### 2.4. Identifying Putative Nodes of Sexual Conflict

In order to significantly reduce the number of false positives among putative nodes of conflict, we took an ultra-conservative approach in defining indirect (unbiased node interacting with sex-biased node) and direct (male node interacting with female node) nodes of conflict. All sex-biased nodes that may potentially be involved in sexual conflict require a very stringent 32-fold expression difference between sexes. To be labeled as a male node, a gene has to be expressed, on average, 32 times greater in males relative to females. Sex-unbiased genes remain defined as any gene that has less than a two-fold expression difference between males and females.

## 3. Results and Discussion

### 3.1. Male and Female Genetic Networks

In nearly all sexual taxa surveyed, reproductive traits and genes are consistently rank among the most rapidly evolving functional classes (see [2]). Most reproductive genes are sex-specific and play a role in maintaining and promoting the divergence of sexually dimorphic traits over time. Sexual antagonism, or conflict, can provide an evolutionary and molecular mechanism to explain the rapid divergence of reproductive genes on a genome-wide scale. The goal of this present study was to identify interaction targets, using a genomics approach, that may potentially be in conflict with each other. To accomplish this goal, we first generated male, female, and sex unbiased networks in *D. melanogaster* by combining sex-specific gene expression with available curated interaction data.

In total, 12,453 genes (12,628 from the permissive set), representing over three quarters of all known *D. melanogaster* genes, were used in this analysis and 237,954 (403,518 using the permissive criterion) interaction partners were identified.  Table 1 provides a summary of all interaction subnetworks. 1,327 male nodes interacted with another male node (the male-male subnetwork), representing over 10% of the total number of assessed genes. Similarly, 1,348 female nodes interacted with at least another female node (the female-female subnetwork). However, unlike the male network with 1,248 male nodes that included interactions not involving other male nodes, the female network only contained 319 non-female-interacting nodes. This may indicate that the female network contains a much larger fraction of shared subnetworks (e.g., female-unbiased, female-female, and female-male) that are more interconnected relative to the male network. Overall, the sex-unbiased network comprised of a much larger fraction of genes (Table 1); however, this high proportion is partially due to the use a very conservative sex-bias stringency.

**Figure 1:**
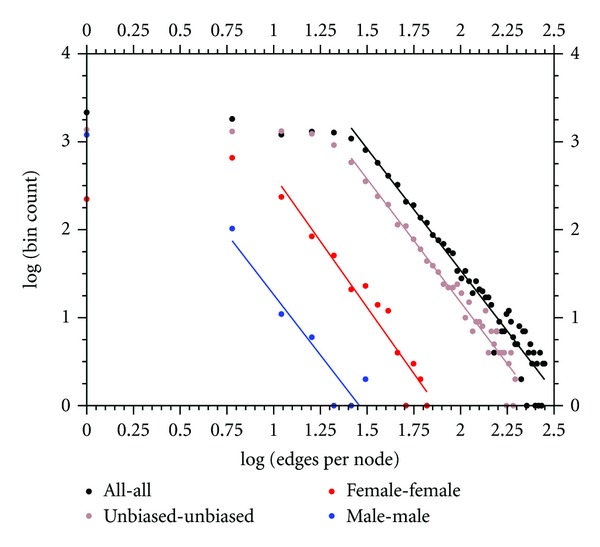
Frequency distribution of the number of edges per node for various classes of interactions. A log-log plot of the edges per node bin counts is shown for four (sub)networks, as indicated in the legend. The bin size is 5, and the bin counts are unnormalized. All interactions from the entire network (black) and those among the sex-unbiased node subnetwork (brown) display power law tails for degree above: 25. The female subnetwork (red) has a power law tail for degree above: 5, while the male subnetwork (blue) is approximately a power law in its entirety. Only the male subnetwork displays a monotonic distribution.

The bin counts on the number of edges per node of the subnetworks, shown in Figure 1, display the often quoted power law behavior of genetic interaction networks, at least on the high degree tails (right tail). Nodes of lower edge/node degree deviate from this scale-free pattern, resulting in a complex network containing at least two distribution behaviors. This observation highlights that care must be taken when fitting degree distributions of genetic interaction networks to power laws. Here, we used a lower cutoff for the degree and only used nodes with number of edges greater than or equal to the cutoff when computing the power law fit. The specific cutoff values, which vary across the subnetwork types, were determined by visual inspection of the distributions, and tend to accord with the average edges per node values in Table 1. If the entire data set is fit to a power law without regard to whether a power law is appropriate over the entire range of node degrees, the resulting best fit power law exponents are problematic to interpret. For example, the female subnetwork shown in Figure 1 (red) displays a peak in the distribution around 5 edges per node. At higher degree values the distribution is approximately a power law with exponent, −3.01 (Table 1). If a cutoff was not used, the resulting power law exponent would be much smaller, −1.52 (data not shown). In some contrast, the male subnetwork, shown in Figure 1 (blue), has a monotonic distribution but tends to deviate from power law behavior at the smallest degree bin size (the distribution flattens out at lower degree values). Beyond the first bin, the best fit power law exponent for the male subnetwork is −2.75. If all the data were used in the power law fit, the resulting best fit exponent is −2.15 (data not shown), which is lower than the power law tail value, as in the female case. The impact of fitting the entire data set, even among very low degree nodes, in the power law fit has the greatest impact on the female subnetwork, and overstates the shallowness of female subnetworks compared to male subnetworks. In fact, both female and male subnetworks have similar power laws in their high degree tails, but differ mainly in the distribution on the low degree end.

Malone et al. [18] recently generated male and female genetic networks in *D. melanogaster*, based on coexpression correlations. In total, they identified 4,104 female-biased genes and 2,694 male-biased genes using a more quantitative approach (our very conservative approach identifies a smaller gene set among male and female networks). Almost 60% of male nodes from our study matched the Malone et al. male network (1,494 out of 2,575). Similarly, there was a nearly 70% concordance among our study's identified females nodes compared to Malone et al.'s female network (1,012 out of 1,446). These overlaps are surprising since each study used very different approaches to assign interaction. Both our studies, report that the female and male subnetworks display a different overall structure. However, while Malone et al. 2012 base their conclusions on different power law exponents for the female and male subnetworks (−1.06 and −1.35, resp.), we find that the difference is not in the power law behavior on the high degree end (which are qualitatively, if not quantitatively similar) but in the deviation from power law behavior at the low degree end. Female subnetworks have a large cluster of genes with: 5–10 interaction partners, while for genes in the male subnetwork, the most frequent number of interaction partners is unity.

This difference between male and female networks, in terms of the identity of their interaction partners, is most easily observed as the total number of interactions that both networks harbor. While the number of female-female interactions, or edges, is an order of magnitude higher than the number of female nodes, male-male nodes have a much smaller number of partners (Table 1). This pattern can also be seen in the frequency distribution of the number of edges (Figure 1). The male degree distribution peaks at its lowest value (a single edge), while the female distribution peaks at 28 edges, more similar to the distribution of all genes without regard to their sex-bias. In other words, male-biased genes appear to be far less interconnected with each other than similar nodes from the female network.

The less interactive nature of male networks (male-any) and subnetworks (male-male) is supported by evolutionary analyses that characterize new gene formation. These genome-wide analyses find that *de novo* genes are expressed often exclusively in males, and preferentially in the testis [19, 20]. It is possible that these genes generally become immediately coopted into male gametogenesis and fertility functions without embedding themselves into the existing male network. In contrast, female genes are rarely found among new genes and often have functions that overlap with embryogenesis and early development [21]. Furthermore, male-driven sexual selection may provide higher selective pressure to maintain and fix these male genes in populations, relative to female genes [22]. Thus, the interconnectivity of the female versus male networks may be the result of a combination of developmental system constraints and historical selective pressures.

### 3.2. Genome-Wide Sexual Conflict

**Figure 2:**
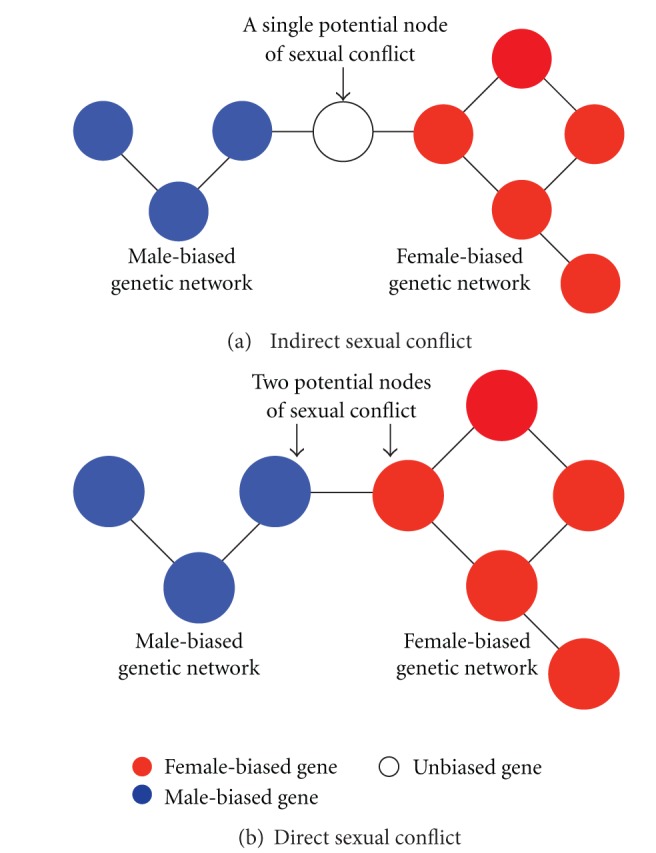
Identifying potential nodes of interlocus sexual conflict from male and female gene networks. Genes (nodes) are classified as male-biased (blue), female-biased (red), and sex-unbiased (open) according to their relative expression levels in each of the sexes. (a) Unbiased genes that are connected to both male- and female-biased genes are potential “indirect” nodes of interest for sexual antagonism at that locus. In other words, indirect sexual conflict can occur in sex-unbiased genes that have edges to both male biased and female biased genes. (b) A sex-biased gene directly connected to a biased gene from the opposite sex provides potential “direct” nodes of interest for sexual antagonism at that locus. In other words, direct sexual conflict can occur on male-biased genes that directly interact with female-biased genes, and vice versa.

By annotating the male and female networks using the unprecedented resources of *Drosophila melanogaster*, we are able, for the first time, to identify putative interacting nodes of conflict, across the genome. To understand the potential extent of genome-wide sexual conflict, we characterized sex-unbiased nodes (i.e., not already part of the reproductive network) that had connections to both highly male and highly female nodes (indirect conflict) as well as candidate nodes under direct conflict, that is, male-female edges (see Figure 2).

Our results supports the contention that sexual conflict has an enormous pool of indirect and direct targets to act extensively upon in the genome. We identified 598 sex-unbiased genes that can potentially act as indirect nodes of interlocus sexual antagonism in addition to 271 direct nodal pairs of potential conflict between male- and female-biased genes. (FlyBase accession lists for indirect and direct, permissive and strict, and male and female networks, are found in Supplementary Files, available on line at http://dx.doi.org/10.1155/2013/545392). A cursory GO analysis of these potentially conflicted sex-unbiased genes identified a variety of development and morphogenesis functional classes that were significantly overrepresented among the sex-unbiased, indirect candidate nodes (Table 2). Since developmental genes are generally more pleiotropic than other genes [23, 24], they may be indirectly involved in various male and female functions including testis and ovary development. It is also possible that different tissue types and developmental stages harbor different interactions. For future studies, it would be important to ensure that interaction datasets are derived from the same tissues and development timepoint as their sex-based expression experimental counterparts. Among the direct male-female conflict candidates, there was a lack of statistically significant gene ontology classes across male genes. However, female-biased genes involved in a direct interaction with a male node contained a range of GO terms with female gametogenesis and reproduction featured prominently (Table 3).

Innocenti and Morrow [25] used a different genome-wide approach to characterize potential nodes of conflict in flies by combining fitness levels of various lines with their gene expression levels, [25]. Specifically, they sampled gene expression levels in males and females across a sample of hemiclonal lines with opposing fitnesses between sexes. Their results identified putative genes involved in sexual conflict (and not the particular gene-pair interactions, as in our work). Overall, their screen found that 8% of all genes may be involved in sexual conflict. We looked for any significant overlap between our putatively conflicted genes and those genes identified by Innocenti and Morrow [25]. There was no significant overlap between our indirect conflict genes, for either the strict or permissive network, and those listed in Innocenti and Morrow [25] (hypergeometric test, two-tailed *P* value ≥0.5). On the contrary, there was a significant lack of overlap between our candidate genes for both the strict and permissive network, and those found in their survey (hypergeometric test, one-tailed *P* value ≤0.01). This suggests that there are other classes of epistatic interactions that have the potential to harbor conflict dynamics.

From these two complementary studies, it appears that the genome provides a potentially large arena to precipitate an extensive evolutionary arms race between the sexes. However, while intuitively appealing, sexual conflict represents just one theoretical perspective to explain such sexual selection phenomena as rapidly evolving reproductive traits and genes, exaggerated sexual characters, and hybrid incompatibility [26]. Catalyzed by large variances in reproductive success, sexual selection can also be explained by alternative coevolutionary processes. Civetta and Singh suggest that sexual traits (and by extension, genes) can evolve rapidly under a process of sexual coadaptation that would harbor a different evolutionary dynamic including greater intraspecific variation [27]. Further work using population and interspecific variation may shed light on these alternative hypotheses.

## 4. Concluding Remarks

The recent availability of genome-wide datasets has unveiled an unprecedented opportunity for biologists to explore the genomic landscape in a systematic manner. By combining whole genome transcriptomes from males and females with genome-wide genetic interactions, we can begin to understand the genetic architecture of sexual dimorphism. With male and female networks identified in *D. melanogaster*, we are well on our way to understanding their evolution, and the evolution of potentially conflicted genes across populations and in other fly species. From a network perspective, it may be more difficult for a female-biased gene to evolve rapidly because of her greater number of interacting partners (i.e., greater selective constraints). Accordingly, we see evidence of lower levels of female-specific gene divergence compared to male-specific gene divergence in multiple studies from protein gel electrophoresis [28] to genomic [22, 29]. Applying a network approach can help move evolutionary genetics from out of its “beanbag” stage [30] and provide us with a new way to understand rapidly evolving gene networks and reproductive systems as a whole.

## Supplementary Material

The supplementary material contains nine data files, described as follows:
“direct_sexconflict_f_permissive.txt” lists gene IDs for the very female biased genes with an edge to a very male biased gene, based on the permissive network.
“direct_sexconflict_m_permissive.txt” lists gene IDs for the very male biased genes with an edge to a very female biased gene, based on the permissive network.
“direct_sexconflict_f_strict.txt” lists gene IDs for the very female biased genes with an edge to a very male biased gene, based on the strict network.
“direct_sexconflict_m_strict.txt” lists gene IDs for the very male biased genes with an edge to a very female biased gene, based on the strict network.
“gene_bias.txt” lists gene IDs and the gene sex-bias bias value. All genes with non-zero expression are included. Note that not all these genes will be in the networks.
“indirect_sexconflict_permissive.txt” lists gene IDs for the unbiased genes that have both male biased and female biased network neighbors, for the permissive network.
“indirect_sexconflict_strict.txt” lists gene IDs for the unbiased genes that have both male biased and female biased network neighbors, for the strict network.
“ppi_permissive.txt” is a two column file of the gene ID pairs that are connected by an edge in the permissive network.
“ppi_strict.txt” is a two column file of the gene ID pairs that are connected by an edge in the strict network.

## Figures and Tables

**Table 1:** Characteristics of sex-biased and unbiased networks. Each of the sex-unbiased, male-, and female-biased networks are classified into subnetworks by the expression bias of the interacting nodes. The letter codes A, U, F, and M refer to the node types “All”, “Unbiased”, “Female-biased”, and “Male-biased”, respectively. The number of nodes and the number of edges refer to first letter (boldface) of the network type. For example, the pair ** X**-Y refers to all nodes of type X that have an edge to a node of type Y. The summary statistics reported are: (1) the total number of nodes (of boldface type), (2) the total number of unique edges connecting those nodes, (3) the overall ratio of edges per node, computed by dividing the second column by the first column, (4) the mean number of edges per node, (5) the standard deviation in the number of edges per node about the mean, (6) the 25th percentile of the number of edges per node, (7) the 75th percentile of the number of edges per node, (8) the exponent of the best fit power law to the degree distribution, and (9) the degree lower limit cutoff for a node to be included in the power law fit. Reported values are for the strict DroID network that only include interactions based on physical, experimental evidence; values in parenthesis refer to the permissive network that include all evidence types in the DroID database version “2012_04”. The power law exponent is fit to the tail of the edges per node binned distribution (bin size = 5); edge per node values below the cutoff indicated in the last column are not used in the power law fit.

Network	Nodes	Edges	Edges/nodes	Mean edges/node	StD edges/node	25th%	75th%	Exponent	Cutoff
** A**-A	12,453 (12,628)	237,954 (403,518)	19.1 (32.0)	38.1 (63.7)	235.0 (244.7)	6 (9)	31 (59)	−2.77 (−2.13)	25 (25)

** U**-A	8,432 (8,517)	219,401 (370,642)	26.0 (43.5)	39.2 (65.6)	233.9 (244.1)	7 (11)	33 (61)	−2.79 (−2.17)	25 (25)
** U**-U	8,302 (8,429)	124,730 (214,508)	15.0 (25.4)	36.6 (61.3)	219.9 (229.3)	6 (10)	30 (57)	−2.80 (−2.19)	25 (25)
** U**-F	7,378 (7,680)	68,794 (108,317)	9.3 (14.1)	31.1 (51.6)	197.5 (206.0)	5 (7)	26 (47)	−3.13 (−2.46)	5 (5)
** U**-M	6,024 (6,802)	25,877 (47,817)	4.3 (7.0)	27.3 (44.7)	183.5 (190.3)	3 (5)	24 (40)	−2.61 (−2.66)	5 (5)

** F**-A	1,446 (1,452)	85,430 (137,027)	59.0 (94.4)	28.6 (46.7)	192.8 (199.4)	3 (5)	25 (41)	−2.59 (−1.54)	25 (25)
** F**-U	1,430 (1,440)	68,794 (108,317)	48.1 (75.2)	29.2 (47.5)	196.5 (202.9)	3 (5)	25 (42)	−2.67 (−1.73)	20 (20)
** F**-F	1,348 (1,365)	9,270 (16,956)	6.9 (12.4)	28.7 (46.9)	193.9 (200.3)	4 (5)	24 (41)	−3.01 (−1.92)	10 (10)
** F**-M	1,127 (1,244)	7,366 (11,754)	6.5 (9.4)	28.2 (45.9)	191.7 (198.0)	3 (5)	24 (40)	−2.09 (−2.23)	5 (5)

** M**-A	2,575 (2,659)	35,160 (63,737)	13.7 (24.0)	27.5 (44.9)	188.3 (194.7)	3 (4)	23 (39)	−2.55 (−1.79)	15 (15)
** M**-U	2,423 (2,547)	25,877 (47,817)	10.7 (18.8)	26.7 (43.7)	184.8 (191.2)	3 (4)	23 (38)	−2.83 (−1.98)	15 (15)
** M**-F	1,622 (1,832)	7,366 (11,754)	4.5 (6.4)	26.1 (42.5)	182.1 (188.2)	3 (4)	22 (37)	−2.90 (−2.42)	5 (5)
** M**-M	1,327 (1,636)	1,917 (4,166)	1.4 (2.5)	25.5 (41.4)	180.0 (185.7)	3 (4)	22 (36)	−2.75 (−2.18)	5 (5)

**Table 2:** Significant gene ontology (GO) categories for sex-unbiased nodes that interact with at least one male-biased gene and at least one female-biased gene (indirect nodes of sexual conflict). Interactions using the strict criterion were used and only significant (Bonferroni corrected *P*-values ≤0.05) gene ontology classes (Biological Process “Fat” as computed in DAVID) that contain at least 10% of the gene set, are listed.

Rank	Gene ontology function (BP)	Gene ontology ID
1	Post-embryonic morphogenesis	GO: 0009886
2	Imaginal disc development	GO: 0007444
3	metamorphosis	GO: 0007552
4	Pattern specification process	GO: 0009653
5	Post-embryonic development	GO: 0009791
6	Regulation of RNA metabolic process	GO: 0009887
7	Instar larval or pupal development	GO:0002165
8	Regionalization	GO: 0003002
9	Regulation of transcription, DNA-dependent	GO: 0006355
10	Regulation of transcription	GO: 0045449
11	Reproductive cellular process	GO: 0048610
12	Gamete generation	GO: 0007276
13	Sexual reproduction	GO: 0019953
14	Reproductive process in a multicellular organism	GO: 0048609
15	Multicellular organism reproduction	GO: 0032504

**Table 3:** Significant gene ontology (GO) categories for female-biased genes that interact directly with male-biased genes (direct nodes of sexual conflict). Interactions using the strict criterion were used and only significant (Bonferroni corrected *P* values ≤0.05) gene ontology classes (Biological Process “Fat” as computed in DAVID) that contain at least 10% of the gene set, are listed. Male-biased nodes of direct conflict did not harbor any significant GO terms.

Rank	Gene ontology function (BP)	Gene ontology ID
1	Notch signaling pathway	GO: 0007219
2	Sexual reproduction	GO: 0019953
3	Female gamete generation	GO: 0007292
4	Reproductive cellular process	GO: 0048610
5	Gamete generation	GO: 0007276
6	Reproductive process in a multicellular organism	GO: 0048609
7	Multicellular organism reproduction	GO: 0032504
8	Cell fate commitment	GO: 0045165
9	Cell fate specification	GO: 0001708
10	Sensory organ development	GO: 0007423
11	Oogenesis	GO: 0048477
12	Cell fate determination	GO: 0001709
13	Reproductive developmental process	GO: 0003006

**Table 4:** 

FRIEDMANPERRIMON_COAP.txt	RNA_GENE.txt
CURAGEN_YTH.txt	FLY_GENETIC_INTERACTIONS.txt
HYBRIGENICS_YTH.txt	F LY_GENE_ATTR.txt
DPIM_COAPCOMPLEX.txt	HUMAN_INTEROLOGS.txt
FLY_OTHER_PHYSICAL.txt	YEAST_INTEROLOGS.txt
TF_GENE.txt	WORM INTEROLOGS.txt
	confidence_correlation.txt
